# Improved insulin injection technique, treatment satisfaction and glycemic control: Results from a large cohort education study

**DOI:** 10.1016/j.jcte.2020.100217

**Published:** 2020-02-04

**Authors:** Malgorzata Gorska-Ciebiada, Malgorzata Masierek, Maciej Ciebiada

**Affiliations:** a Department of Propaedeutics of Lifestyle Diseases, Medical University of Lodz, Poland; b Bioton S.A., ul. Starościńska 5, 02-516 Warsaw, Poland; c Department of General and Oncological Pneumology, Medical University of Lodz, 22 Kopcinskiego Street, 90-153 Lodz, Poland

**Keywords:** Insulin injection technique, Education, Glycemic control

## Abstract

**Objective:**

The aim of this study was to elucidate injection techniques, treatment satisfaction and glycemic control after education among patients with type 2 diabetes.

**Methods:**

4513 insulin-treated diabetic patients enrolled in the observational study EGIDA II (Education and GensuPen In Diabetology II) filled out the questionnaire which focused on key insulin injection parameters, pain sensation scale and satisfaction of the treatment form before (visit 1) and after 3 months treatment with insulin injection (visit 2). The education was performed by trained healthcare professionals. To assess the utility and comfort during using new automatic injection system (GensuPen) we separated 2 groups: A – treated with GensuPen and B – treated with other pens.

**Results:**

The education resulted in increased number of patients who properly remix cloudy insulin; inject insulin into skin; change every time the injection site; use the pen needle only once; prepare a pen for injection and store insulin. We noticed significant decrease in BMI and sensation of pain in both groups. Our study revealed that patients’ satisfaction with the treatment increased with each of the 5 items (type of the treatment, mood, physical activity, vital energy, a sense of control over the disease) using a 5-point scale, with greater increase in group A. The utility and comfort (weight, thickness, easiness in remove pen cap, cleaning, twisting, keeping in hand the pen, dial the dose, readable signaling of injected dose) during using the GensuPen significantly increased in group A. Finally the mean glucose level in self-control diary was significantly lower after 3 months of the treatment in both groups, however the difference between visit 1 and 2 was greater in group A.

**Conclusion:**

The study showed that proper selection of pen and professional education can result in the improvement of insulin injection technique, higher patients’ satisfaction and better glycemic control.

## Highlights

• The efficacy of insulin therapy in diabetes depends on proper injection technique.
• Professional education can results in higher patients’ satisfaction.
• Proper insulin injection is important to good glycemic control.

## Introduction

Type 2 diabetes (T2DM) represents a global health problem with the heavy socio-economic burden. This chronic disease is predicted to affect approximately 415 million adults by 2015 and almost 642 million adults by 2040 [1]. It caused around 5 million deaths in 2015 and up to 1,197 billion dollars were spent due to diabetes worldwide [1]. T2DM is associated with severe complications (e.g. cardiovascular disease, kidney failure, blindness, lower limb amputations) which can cause a death or shorten patients’ life with lowering quality of life [2], [3]. The pathogenesis of T2DM is multifactorial and the treatment often includes many oral antidiabetic or hypoglycemic agents often with/or insulin injections. Polish Diabetes Association as well as European and US guidelines recommend insulin as an option after life style modification and metformin to bring glycated haemoglobin (HbA1c) below a general target of 7% (53 mmol/mol) [4], [5], [6].

There is a strong evidence that proper use of insulin injection technique is crucial for optimizing the efficacy of the therapy. Many recommendations published by different diabetes associations are based on the results of Injection Technique Questionnaire (ITQ), which is one of the largest multinational studies of this kind [7]. Although it is obvious that proper insulin injection is important to good glucose control with lowering the risk of diabetes related complications, recent studies showed that only too few patients can understand this problem. In addiction providing better devices with many modern solutions can result in higher effectiveness of insulin therapy. GensuPen - new automatic injection system has many advantages over other pens such as: constant, optimal speed of insulin delivery, conveniently located insulin release button, the lower minimum strength needed to make the injection, diversified sound when adjusting and withdrawing the dose, an additional injection rate indicator, and easy dose correction without losing insulin. GensuPen is intended to deliver any type of insulin: short or long acting regular insulin or premixed human 30/70, 40/60 or 50/50 insulin. There is a great emphasis on the education and training of patients who are treated with insulin injections. In polish diabetic population data about insulin injection technique are poor. It is important to know exactly how patients inject as a prerequisite for further education and better glycemic control. Therefore the aim of this study was to elucidate insulin injection techniques, treatment satisfaction and glycemic control after education among patients with type 2 diabetes in large cohort of polish population.

## Material and methods

This study was a 12-week, multicenter, observational trial - EGIDA II (Education and GensuPen In Diabetology II). The population of 4513 insulin-treated patients with type 2 diabetes was enrolled in the study according to the study protocol. All subjects were recruited from outpatient diabetology clinics localized in different parts of Poland. This study wasn't designed as a randomized controlled trial. To eliminate selection bias, eligible and consenting patients were accessioned consecutively as they entered the clinic. The inclusion criteria were as follows: diagnosis with type 2 diabetes; age 18 years or older; the general state (psychophysical) health of the patient, adhering to the medical recommendations; treatment with insulin for at least 12 months. The doctors participating in the study based on patient preference, made an independent decision about replacing a manual insulin pen into new automatic injection system (GensuPen) (Group A) or staying with your current manual insulin pen (Group B). Subjects with psychiatric disorders, addiction to alcohol and drugs, allergy to insulin or any of the components of the preparation, newly diagnosed with type 2 diabetes and/or with lastly beginning with insulin injections treatment; patients with the impairment of the dominant hand, which makes it impossible to operate the insulin pen individually; were excluded from the study.

The study consisted of a well-structured questionnaire used for data collection that is divided into three parts. First part of the questionnaire was performed by specialists – diabetologists (visit 1) and included general questions about the patient’s demographics, duration of diabetes and insulin use, type of insulin and number of units, Hba1c level from previous three months before visit, mean glycaemia level from self-control diary, episodes of severe hypoglycemia during last 12 weeks. In addition this section included a patients’ satisfaction of the treatment form. This form included 5 items (type of the treatment, mood, physical fitness, vital energy, a sense of control over the disease) using a 5-point Likert scale of extremely satisfied = 5 points, and extremely dissatisfied = 1. The second part of the questionnaire was performed by trained healthcare professionals – educator nurse and included 33 questions highlighting the administration techniques and also pain sensation scale. The pain sensation scale includes 10 points where 0 point means no pain and 10 points means the highest pain during insulin injection. The questionnaire was followed by detailed education performed by trained healthcare professionals at the centers. The third part of the questionnaire was performed by educator nurse after 12 ± 2 weeks with insulin injection (visit 2) and included questions highlighting the administration techniques, pain sensation scale and a patients’ satisfaction of the treatment form.

All questionnaires had been originally prepared for the EGIDA II study. The Authors had planned to recruit subjects and divide the pool of patients into two study groups, in a ratio of 5 to 1. Hence, assuming α = 0.001 along with a level of statistical power at 0.95 (in order to achieve a very high precision and reliability of measurements), the required minimum sample size amounted to, respectively, n = 3611 and n = 723 persons.

All continuous results were described as means ± standard deviation values whereas all categorical data were presented as absolute numbers and percentages. The normality of distribution was verified by using the Shapiro-Wilk W test. Descriptive statistics for the categorical variables were assessed using the χ^2^ and for the continuous variables using Student’s *T*-test, paired and unpaired, or the Mann Whitney *U* test and Wilcoxon signed-ranks test when applicable. Repeated observations with discrete variables were analyzed by using the marginal homogeneity test or McNemar’s χ^2^ test (for binomial traits only). Relationships were investigated by using Pearson’s product-moment correlation analysis for normally distributed variables and Spearman’s rank correlation coefficient for non-normally distributed or ordered variables. A P value of <0.05 was considered statistically significant; a two-side approach was implemented. Missing data were case-wise deleted according to each particular procedure. Stata/SE 12.1 (StataCorp LLC, College Station, Texas, USA) was used for the analyses.

All procedures followed were in accordance with the Declaration of Helsinki, and the study protocol was approved by local ethical review boards (Medical University of Lodz, Poland). All study participants were informed about the purpose of the study and additional information was given as they need. Written informed consent was obtained from all patients for being included in the study.

## Results

### Baseline demographic and clinical characteristic

The demographic and clinical characteristics of the study group have been presented in Table 1. According to the study design 4513 patients were dived into 2 groups: group A – treated with GensuPen included 3765 subjects and group B – treated with other pens included 748 subjects (this refers to the Full Analysis Set, FAS, regardless the missing data which randomly occurred). The mean age of the study participants was 65.3 ± 10.2 years, mean BMI was 30.4 ± 5.2 kg/m2, mean diabetes duration was 10.3 ± 6.8 years, mean insulin treatment duration was 5.4 ± 5.0 years and mean baseline HbA1c was 8.2%  ±  1.5%. The study groups didn’t differ in age, sex distribution, BMI, diabetes duration, HbA1c level.

**Table 1:** Demographic and clinical characteristics of type 2 diabetic patients treated with insulin injections.

	All subjects	Group A	Group B	P value
No of patients (FAS)	4513	3765	748	
Age (years)	65.3 ± 10.2	65.4 ± 10.2	64.7 ± 9.9	p = 0.096
Sex, female	2381 (53.8%)	1997 (53.9%)	384 (52.9%)	p = 0.611
BMI (kg/m^2^)	30.4 ± 5.2	30.4 ± 5.3	30.1 ± 4.7	p = 0.150
Duration of diabetes (years)	10.3 ± 6.8	10.1 ± 6.7	11.4 ± 6.9	p < 0.001
duration of insulin treatment (years)	5.4 ± 5.0	5.2 ± 4.9	6.3 ± 5.7	p < 0.001
HbA1c level	8.2% ± 1.5%	8.3% ± 1.5%	8.0% ± 1.4%	p < 0.001
mean glycaemia level from self-control diary (mg/dl)	170.2 ± 41.9	171.4 ± 42.6	159.4 ± 33.1	p < 0.001
Total daily dose of insulin (IU)	42.7 ± 20.0	42.6 ± 20.1	43.3 ± 18.4	p = 0.378
Type of insulin				
Short-acting insulin (%)		1321 (35.1%)	118 (15.8%)	
Long-acting insulin (%)		1195 (31.7%)	107 (14.3%)	
Mean dose of insulin (IU)				
before breakfast	20.6 ± 8.5	20.7 ± 8.5	19.4 ± 8.3	p < 0.001
before lunch	12.1 ± 6.2	12.2 ± 6.0	11.7 ± 5.5	p = 0.035
before dinner	15.5 ± 7.1	15.6 ± 7.2	14.8 ± 6.8	p = 0.005
before night	15.6 ± 7.1	15.6 ± 7.2	15.9 ± 6.6	p = 0.292
Time of the day of insulin injection				
before breakfast	4117 (91.2%)	3417 (90.8%)	700(93.6%)	p = 0.013
before lunch	1864 (41.3%)	1515(40.2%)	349(46.7%)	p = 0.001
before dinner	3817 (84.6%)	3169(84.2%)	648 (86.6%)	p = 0.089
before night	1562 (34.6%)	1282(34.1%)	280 (37.4%)	p = 0.076
episodes of severe hypoglycemia during last 12 weeks	601(13.2%)	467 (12.4%)	134 (17.9%)	p < 0.001

Values are expressed by mean ± SD or frequency. The Student *t* test, Mann-Whitney *U* test, or chi2 test was used to test for significant differences.

DM2 – diabetes type 2, HbA1c – glycosylated hemoglobin, BMI – body mass index.

In the present study, 4117 (91.3%), 1864 (41.3%), 3817 (84.6%), and 1562 (34.6%) of participants required injection before breakfast, lunch, dinner and night, respectively and there was no difference between groups in these data. The types of insulin pen used in group B included: NovoPen − 447 (59.8%) of patients; HumaPen ERGO – 142 (19%) of patients; HumaPen Luxura HD – 71(9.5%) of patients; SoloStar – 34 (4.5%) of patients and AutoPen – 25 (3.3%) of patients.

### Education and training

The positive effects of education and training in both groups are described in Table 2. We noticed that the education resulted in the improvement of insulin injection technique in parameters mention below. Number of patients who properly remix cloudy insulin (e.g. NPH) increased significantly in both groups (p < 0.001). Subjects more often inject correctly into a lifted skin-fold with proper releasing and at an angle of 90° and keep the pen needle under the skin for >10 s (P < 0.001). Number of patients who change every time the injection site and who use the pen needle only once increased significantly in both groups (p < 0.001). Subjects more often correctly prepare a pen for injection (p < 0.001), and store it properly (p < 0.001). Results show that 8-mm needle lengths are used by nearly 50% of patients and 6-mm needles by approximately 36% and these results didn’t changed after treatment period and education. We noticed significant decrease in BMI in both groups (p < 0.001): group A, visit 1–30.4 (kg/m2), visit 2 – 30.1 (kg/m2); group B, visit 1–30.07 (kg/m2), visit 2 – 29.8 (kg/m2).

**Table 2:** Effects of education and training on administration techniques in type 2 diabetic patients treated with insulin injections.

Number of patients who:	Group A before education (visit1)	Group A after education (visit 2)	Group B before education (visit1)	Group B after education (visit 2)
properly remix cloudy insulin	1878 (51.4%)	2864 (79.2%)*	420 (57.8%)	572 (80.8%*)
inject correctly into a lifted skin-fold with proper releasing and keep the pen needle under the skin for > 10 s	2091 (90.3%)	2488 (98.8%)*	433 (92.5%)	509 (99.6%*)
inject correctly at an angle of 90°	2373 (66.2%)	2525 (70.5%)*	486 (66.8%)	503 (70.9%*)
change every time the injection site	2379 (64.5%)	2964 (80.0%)*	488 (69.0%)	594 (80.9%*)
use the pen needle only once	250 (6.7%)	1068 (28.4%)*	59 (8.0%)	213 (28.8%*)
correctly prepare a pen for injection	1714 (46.1%)	3087 (83.4%)*	388 (52.7%)	614 (83.4%*)
correctly store used insulin	3258 (87.0%)	3513 (93.8%)*	664 (89.9%)	700 (94.3%*)
correctly store unused insulin	3571 (95.0%)	3719 (99.1%)*	721 (96.9%)	741 (99.6%*)

* Difference statistically significant, p < 0.001, visit 1 vs. visit 2.

### Sensation of pain scale

**Fig. 1:**
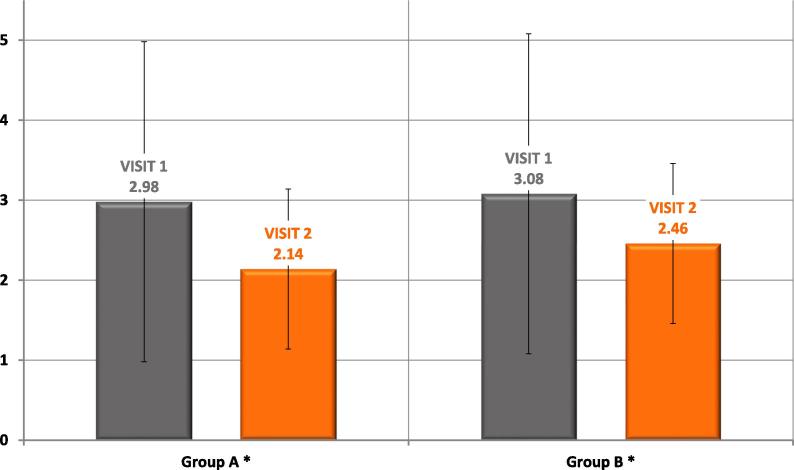
Sensation of pain scale in type 2 diabetic patients treated with insulin injections before (visit 1) and after education (visit 2).

We noticed significant decrease in sensation of pain in both groups (p < 0.001). Data are presented in Fig. 1.

### Patients’ satisfaction of the treatment

**Fig. 2:**
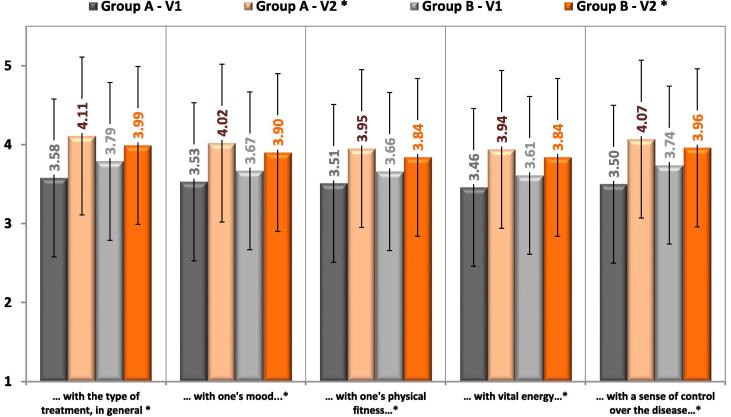
Patients’ satisfaction of the treatment in type 2 diabetic patients treated with insulin injections before (visit 1) and after education (visit 2).

Our study revealed that patients’ satisfaction with the treatment increased with each of the 5 items (type of the treatment, mood, physical fitness, vital energy, a sense of control over the disease) using a 5-point scale, with greater increase in group A, p < 0.001 (Fig. 2).

### The utility and comfort during using new automatic injection system (GensuPen)

In addition we assessed the utility and comfort during using new automatic injection system (GensuPen). We noticed that such parameters as weight, thickness, easiness in remove pen cap, cleaning, twisting, keeping in hand the pen, dial the dose, readable signaling of injected dose during using the GensuPen significantly increased in group A after 3 months of the treatment (p < 0.001) (Table 3).

**Table 3:** The utility and comfort during using new automatic injection system (GensuPen).

Parameter assessed by patient	Group A before education (visit1)	Group A after education (visit 2)	P value
Proper thickness of pen	2912 (77.9%)	3645 (97.2%*)	p < 0.001
Proper weight of pen	2918 (78.0%)	3683 (98.2%*)	p < 0.001
Easiness removing pen cap	2890 (77.6%)	3593 (96.3%*)	p < 0.001
Easiness in cleaning the pen	2442 (66.9%)	2978 (80.5%*)	p < 0.001
Easiness in twisting a pen	2927 (81.9%)	2416 (95.6%*)	p < 0.001
Easiness in keeping a pen in hand	3107 (83.1%)	3622 (97.4%*)	p < 0.001
Easy dial the dose	3256 (87.1%)	3595 (96.4%*)	p < 0.001
Readable signaling of injected dose	2748 (73.7%)	3621 (97.3%*)	p < 0.001

* Difference statistically significant, p < 0.001, visit 1 vs. visit 2.

### Mean glucose level in self-control diary

**Fig. 3:**
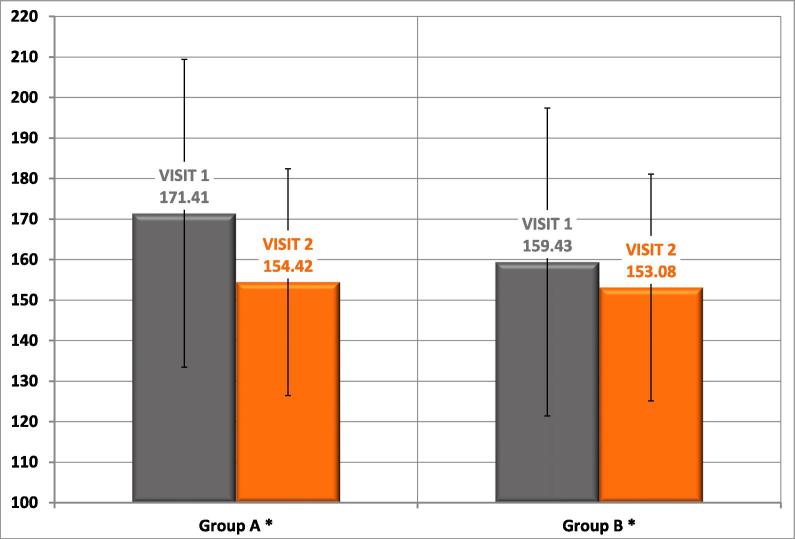
Mean glucose level in self-control diary in type 2 diabetic patients treated with insulin injections before (visit 1) and after education (visit 2).

Finally the mean glucose level in self-control diary was significantly lower after 3 months of the treatment in both groups (group A: visit 1 –171.4 ± 42.6 mg/dl , visit 2 – 154.4 ± 28.6 mg/dl, p < -0.001; group B: visit 1 – 159.4 ± 33.1 mg/dl, visit 2–153.1 ± 28.6 mg/dl, p < 0.001), however the difference between visit 1 and 2 was greater in group A (group A: 16.9 mg/dl, group B: 6.6 mg/dl, p < 0.001) (Fig. 3).

## Discussion

This study performed in large cohort of type 2 diabetic patients was the first in Poland, which described injection techniques, treatment satisfaction and glycemic control before and after education. We noticed that the education resulted in the improvement of insulin injection technique in number of patients who: properly remix cloudy insulin, more often inject correctly into a lifted skin-fold with proper releasing and at an angle of 90° and keep the pen needle under the skin for > 10 s, who change every time the injection site and who use the pen needle only once, correctly prepare a pen for injection and store it properly. Our study indicate that a knowledge of patients how to inject insulin properly is insufficient and requires a professional education. This data are comparable to other studies [7], [8], [9], [10]. The Injection Technique Questionnaire (ITQ) survey [7], which was conducted with 13,289 patients from 423 centers in 42 countries showed also not satisfactory injection practices, e.g. only 31.9% of patients left the needle under the skin the recommended 10 s or longer; whereas 63.7% of patients lifted a skinfold, and 75.0% of these did it correctly.

In our study 8-mm needle lengths are used by nearly 50% of patients and 6-mm needles by approximately 36% and these results didn’t changed after treatment period and education. In ITQ survey the 4- and 8-mm needles are each used by approximately 30% of the total and the 5- and 6-mm needles each by approximately 20% [7]. The needle length can determine intramuscular injection risk which can lead to glucose variability and hypoglycemia [11]. In recent years the needle length has decreased to lower this risk and to reduce anxiety, injection pain and risk of bleeding and bruising. BMI and body site are the most important factors which influence subcutaneous fat thickness [12]. The risk of intramuscular injection increase in men (they have less fat than women for the same BMI), lower BMI and site of injection- thigh or arm. Thus recommendation propose to use shorter needles (4, 5, and 6 mm) by any adult patient, including obese individuals, which should be given at 90° to skin surface and do not generally require the lifting of a skin fold [13].

Much worsen data are referred to changing the needle – in ITQ survey approximately half of the patients worldwide use their needles more than once. In our study only 6.8% of patients before education and only 28.3% after education use the pen needle only once. Reuse of needles can cause the loss of sterility, damage of needle tip or blockage the next dose by residual insulin within the needle, higher risk of pain during injection, even the risk of the needle breaking off and remaining in the tissue [14], [15], [16]. The reason for reuse the needle differs and usually is due to saving money (1/4 of responders), for convenience (e.g. reduction in the need to carry spare needles, avoidance of disposal issues, 41.2% of patients), sometimes to prevent excess waste (environmental concern) [7].

We showed that almost half of subjects properly remix cloudy insulin, and only 46,5% of patients correctly prepare a pen for injection. Incorrect resuspension of cloudy insulin preparations can lead to large dosing errors and influence glycemic control [17]. It is recommended to tip cloud insulins 10 to 20 times to reach stable suspension [18]. Inadequate resuspension of NPH insulin before injection is very common among patients in many studies [7], [17].

Although most of our subjects correctly store used and unused insulin, in ITQ survey 88.6% of subject store insulin in refrigerator, but after opening it, 43.0% continued to store it in the fridge and only 56.3% of these, let it warm up to room temperature before injecting it [7]. Cold insulin is connected to more painful injections.

Another problem is changing every time the injection site, which was performed only by 63.5% patients in our study. Rotation of the injection site is very important to prevent lipohypertrophy (LH). This is the most common skin complication of insulin therapy which affects almost 50% of diabetic patients [19]. LH can significantly reduce the absorption of insulin by up to 25% and thus could worse diabetes control [20]. In contrast to our study a number of patients who rotate sites each time they injected is reported to be different in other countries: 92% (China) [21]; 88.5% (Canada) [22] and only 38% (7 european countries) [23]. In the Injection Technique Questionnaire (ITQ) survey 83.9% of patients claimed to rotate injection site, however 70.6% of these did it correctly [24]. Correct injection site rotation means injecting at least 1 cm from a previous injection. These data show the need for education how to avoid lipohypertrophy. American guidelines for diabetes educator recommend to teach individuals who are self-injecting medications to inspect the intended injection site prior to injection by looking and feeling for hardened areas; to understand the need for regular site rotation and to avoid injecting into areas of LH, inflammation, edema, scar tissue, moles or infection [25]. In our study a number of patients who rotate injection site increase to almost 80%.

Pain is another problem reported by patients. In ITQ survey more than half of the subjects had painful injections, usually connected with bleeding [24]. Pain was also associated with: injecting through clothes, injecting cold insulin, LH, injecting into LH, incorrect site rotation, hypoglycemia and hyperglycemia, higher HbA1c levels, lower BMI, younger age, and higher doses of insulin [24]. In our study we noticed significant decrease in sensation of pain in both groups and thus could be explained by improved injection technique after education, with greater decrease in group treated with GensuPen.

Our study revealed that patients’ satisfaction with the treatment increased with each of the 5 items (type of the treatment, mood, physical fitness, vital energy, a sense of control over the disease) using a 5-point scale, with greater increase in group treated with GensuPen. Perhaps this is connected to effective education, but also it is due to good parameters of new automatic injection system as weight, thickness, easiness in remove pen cap, cleaning, twisting, keeping in hand the pen, dial the dose, readable signaling of injected dose. Diabetic treatment satisfaction could be associated with blood glucose control. We demonstrated that the mean glucose level in self-control diary was significantly lower after 3 months of the treatment in both groups with greater difference between visit 1 and 2 in group A., using GensuPen. This observation is comparable to other studies which showed that greater patients’ satisfaction with the treatment raises the quality of care, improve glycemic outcome and control costs [26], [27], [28].

Both better blood glucose control and treatment satisfaction could be associated with decreased BMI (small but statistically significant) observed in both groups. Although our primary aim was the education in proper injection techniques, the health professionals also provide basic information on the importance of lifestyle changes (healthy diet, physical activity), the importance of home blood glucose monitoring and other topics. Thus possible explanations of weight loss include alterations in body composition, greater motivation of educated patients to maintain or lose weight, alterations in insulin absorption, action and or disposal, and greater patient compliance.

The advantage of this study is that patients got treatment that is the most suitable for them and we obtain data from routine clinical practice. We presented “real world” results of patient-centered approach which include impact on education, self-management and proper treatment with insulin. Whereas randomized control trials showed a methodical approach with defined patient populations, “real life” data obtained in observational studies provide important information into day-to-day medical practice in unselected patient populations.

Our study has also some limitations. Firstly all questions although were prepared according to previous studies, they are not exhausting the subject – for instance we omitted injections complications as lipohypertrophy and recommendation for routine inspection of injection site by health care professionals which is associated with lower glycated hemoglobin levels, more correct injection site rotation and lower risk of lipohypertrophy. Secondly we used the mean glucose level in self-control diary to asses glycemic control. Although these data contribute to chronic glycemia, HbA1c level before and after education could be useful marker. Thirdly although we noticed that the interventions in Group A include switching to GensuPen and providing education, it is not clear if the improvement (from visit 1 to 2) is attributable to the education, or patients’ own learning to use the new pen. It is possible that apart of education, the advantages of new device gave such improvement, therefore it could be interesting to do further prospective observational study only in Group A before and after education staying with new pen.

Despite some limitations, we believe that our data are of high clinical importance. This study gave some useful insight into of injection practices in large cohort of polish diabetic population and showed the importance of education resulted in better patients’ satisfaction and glycemic control.

## Conclusions

The study showed that proper selection of pen and professional education can results in the improvement of insulin injection technique, higher patients’ satisfaction and better glycemic control. Insulin administration remains one of the crucial elements of the patient education. Our study showed that knowledge and practical skills of diabetic patients are insufficient which confirm a need for further education seen as a lifelong process with regular repetition.

## Conflicts of interest

Małgorzata Masierek is an employee of BIOTON S.A. The other authors declare that there is no conflict of interest regarding the publication of this paper.

## Funding statement

The study was funded by BIOTON S.A. BIOTON S.A. was the originator of the study and also the sponsor of the research tool, and publications on the study results.
